# The Effects of a Single Versus Three Consecutive Sessions of Football Training on Postprandial Lipemia: a Randomized, Controlled Trial in Healthy, Recreationally Active Males

**DOI:** 10.1186/s40798-019-0212-1

**Published:** 2019-08-22

**Authors:** Darren J. Paul, Jens Bangsbo, Anissa Cherif, George P. Nassis

**Affiliations:** 1 Aspetar – Qatar Orthopaedic and Sports Medicine Hospital, Scientific Support and Research, PO BOX 29222, Doha, Qatar; 2 Department of Nutrition, Exercise and Sports, Copenhagen Centre for Team Sport and Health, University of Copenhagen, Copenhagen, Denmark; 3 Anti Doping Laboratory of Qatar (ADLQ, Research Department), Doha, Qatar; 4 Department of Sports Science, City Unity College, 10562 Athens, Greece; 5 School of Physical Education and Sports Training, Shanghai University of Sport, Yangpu District, Shanghai, China

**Keywords:** Triglycerides, Post-meal, Team sports

## Abstract

**Background:**

Exercise frequency is important for maintaining health; however, its effects on postprandial responses remain largely unknown. Better understanding this during popular sports activities such as football may influence exercise habits. Therefore, the aim of the present study was to examine the effects of playing one single versus three consecutive days of 60-min small-sided football matches on postprandial lipemia.

**Methods:**

Fifteen males performed either one (1FOOT; *n* = 7) or three 60-min football (3FOOT; *n* = 8) sessions across an 8-day trial period. On day 1, a blood sample was collected at fasted (0 min) and 0.75, 2, 4, 6 h after a high-fat meal. Participants were then randomly allocated to the 1FOOT (day 7) or 3FOOT (days 5, 6, 7) condition. On day 8, they repeated the high-fat meal and blood sampling for 6 h following the meal. Postprandial total and incremental area under the curve (AUC, iAUC, respectively) were calculated.

**Results:**

The postprandial triglyceride iAUC was 41% lower from pre- to post-measures for the 1FOOT (*p* < 0.05; ES = 1.02) and 15.7% lower for the 3FOOT (ns; ES = 0.41). Total triglyceride AUC was lower (26%) post-football matches in the 3FOOT group only (*p* < 0.01; ES = 1.23). In 3FOOT, insulin concentration was lower for post- compared to pre-measures at 0.75 and 2 h, respectively (*p* < 0.001).

**Conclusion:**

One single 60-min small-sided football match lowered postprandial TG incremental area under the curve while performing three consecutive days of football matches did not result in a greater attenuation.

**Trial Registration:**

ISRCTN17934193, registered 06 April 2019

## Key Points

- One single 60-min small-sided football match lowered postprandial TG incremental area under the curve while we did not find a greater benefit to three consecutive days’ exercise on postprandial TG attenuation
- There is no dose-response effect for accumulating consecutive days of exercise on lowering postprandial TG.
- Performing three consecutive days of 60-min small-sided football matches significantly reduced the early phase (0.75 and 2 h) postprandial insulin response.

## Background

In many developed countries, individuals have frequent access to food during waking hours; hence, most of the diurnal period is spent in a postprandial (fed) state [1]. The postprandial increase in triglyceride (TG)-rich lipoproteins (chylomicrons and very low-density lipoproteins) has shown to be associated with atherosclerosis and increase the risk of cardiovascular disease, possibly due to the concomitant inflammatory response and induced oxidative stress [2].

An acute bout of endurance, resistance, and high-intensity exercise have all shown to independently lower postprandial TG [3]. However, these may not be the chosen exercise habits of many individuals. Football, for instance, is a sport played recreationally by millions of people of different age, gender, and pathological state. There has shown to be a broad spectrum of health benefits from playing football [4]. For instance, decreased fasted TG has been reported for a group of overweight adolescents, following a 12-week period of football training three times per week [5]. However, a large body of evidence suggests that postprandial TG rather than fasted measures are a better proxy of lipid metabolic health, since it more closely correlates with cardiovascular disease [6, 7].

Evidence has shown an intermittent football simulation protocol to be as effective as continuous running for lowering postprandial TG in a group of children [8] and young adults [9]. In addition, we found that a 9-a-side football match can lower postprandial TG measures in normal as well as overweight individuals [10], while Smallcombe et al. [11] recently reported a small-sided football match resulted in a similar moderate reduction of postprandial TG in adolescent boys, compared to continuous treadmill running. However, these studies have solely examined the effects of an acute bout of football activity. Since regular exercise is important for maintaining health benefits, it would be worthwhile investigating whether a short period of consecutive days of exercise may elicit greater benefits in postprandial TG.

Exercise-induced energy expenditure is considered an important modulator of the postprandial TG attenuation with this reduction shown to be directly proportional in a dose-dependent manner (up to ~ 800 kcals) [12]. Both a diet- and exercise-induced energy deficit of a similar magnitude (~ 470 kcals) has shown to lower TG concentrations in both the fasting and postprandial states [13]. However, the reduction in the postprandial state was greater after exercise than after energy restriction, highlighting the unique benefits of exercise on lipid metabolism [13]. The amount of energy expenditure can be mediated by several factors; thus, researchers have examined how acute exercise timing, type, and intensity may impact the postprandial TG response [1]. The effects of exercise frequency on postprandial TG, however, are far less understood, possibly due to the supposed transient nature of exercise on lipid metabolism. A study by Farah et al. [14] reported three consecutive days of exercise (walking on a treadmill at an intensity of 50%VO_2_max) does not magnify the postprandial TG attenuation any greater than 1 session following consumption of an ad libitum meal, in a group of sedentary overweight males. Nevertheless, there remains a general dearth of research pertaining to the effects of exercise frequency on postprandial TG, which would be important since the weekly frequency and general exercise characteristics will differ among individuals. It is worth examining whether a greater energy expenditure accumulated over consecutive days would elicit a more profound effect on lowering postprandial TG. Therefore, the aim of the present study was to examine the effects of playing one single versus three consecutive days of 60-min small-sided football matches on postprandial lipemia.

## Methods

### Participants

Fifteen males performed either one (1FOOT; *n* = 7) or three (3FOOT; *n* = 8) 60 min football sessions (participant characteristics presented in Table 1). Exclusion criteria were (1) cardiovascular, pulmonary, or metabolic disease, (2) contraindications to exercise testing as established by the American College of Sports Medicine, and (3) dietary restrictions regarding the meal provided. Participants had previous experience playing football, were recruited from the local recreational football league, and participated in the experiment during a break in the season. Written consent was obtained following detailed instructions by the principal investigator. The study was carried out in accordance with the guidelines in the Declaration of Helsinki and was approved by the local Ethics Committee (Anti-Doping Laboratory Qatar, F2015000112).

**Table 1:** Demographic details of the 1FOOT (one football session) and 3FOOT (three consecutive football sessions) groups (data presented as mean and SD)

	1FOOT (*n* = 7)	3FOOT (*n* = 8)
Age (years)	28.2 (4.1)	31.8 (4.0)
Body mass (kg)	79.1 (8.6)	83.6 (13.6)
W:H ratio	0.81 (0.06)	0.86 (0.07)
BMI (kg/m^2^)	24.4 (2.8)	26.2 (3.4)
YYIETL1 (m)	1194 (396.9)	1235 (491.5)

*W:H ratio* waist to hip ratio, *BMI* body mass index, *YYIETL1* Yo-Yo Intermittent Endurance Test Level 1

### Experimental Design

**Fig. 1:**
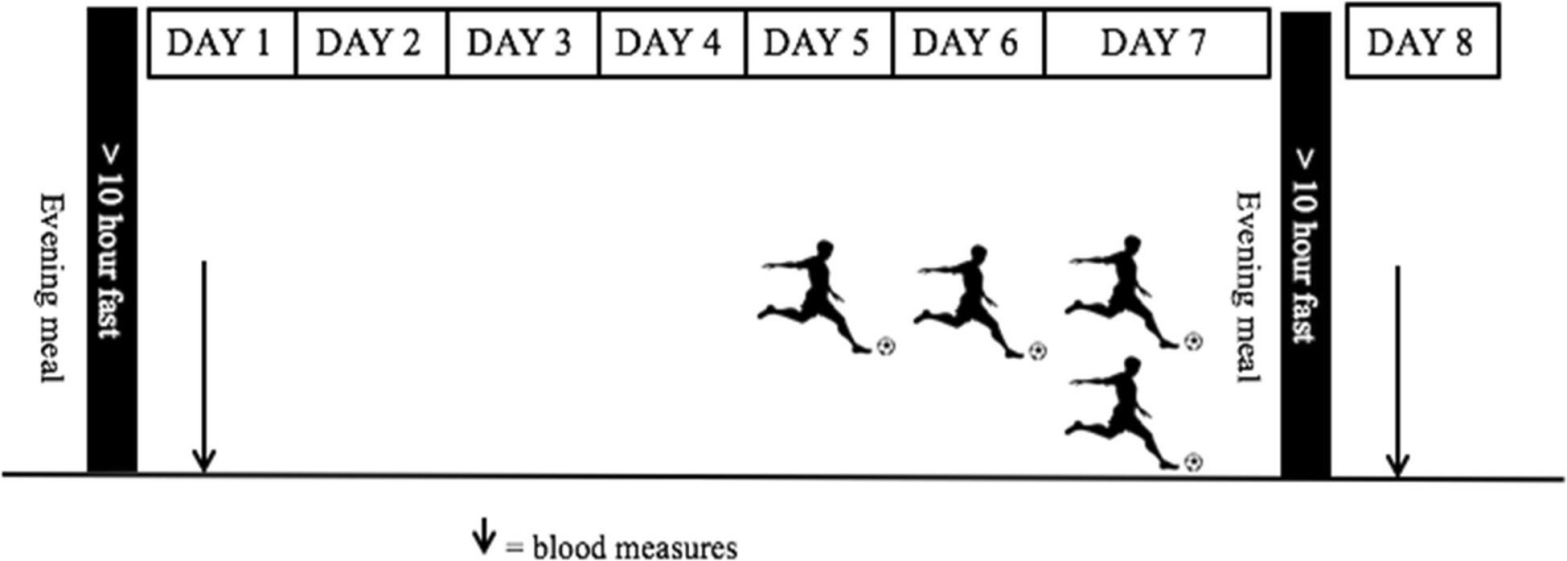
Schematic representation of the study’s design

Participants were randomly allocated to either 1 day (1FOOT; day 7) or 3 days (3FOOT; days 5, 6, 7) of football matches (Fig. 1). To control for confounding variables, participants were instructed to (1) fast for > 10 h before arrival to the laboratory for the high-fat meal consumption and subsequent blood sampling, (2) record and replicate the same dietary intake for 24 h before the laboratory procedure, (3) abstain from caffeine and dietary supplements for 24 h before the laboratory procedure, and (4) be awake between 06:00 and 07:00 h prior to each laboratory trial. Participants were instructed to abstain from exercise training for 1 week prior to testing.

### Preliminary Measures

Body mass and height were recorded using a combined scale and stadiometer (SECA 769, Fremont, CA, US) with body mass index calculated. Waist and hip circumferences were measured at the visual narrowing of the waist between the iliocristale and 10th rib landmarks (waist) and the girth of the buttocks at the level of the posterior protuberance. Body fat was estimated using the InBody Bioelectrical Impedance Analysis System (Inbody 720, Biospace, Seoul, Korea).

Participants performed the Yo-Yo Intermittent Endurance Test Level 1 (YYIETL1), in an air-conditioned sports hall on artificial turf to establish maximal heart rate (HR) and cardiorespiratory fitness. The YYIETL1 test consists of 2 × 20 m shuttle runs performed at increasing running speeds, interspersed with 5 s of active recovery, during which the participants jogged around a cone placed 2.5 m behind the start/finishing line. The speeds were controlled by audio and the test was terminated when the participant was no longer able to maintain the required speed for two consecutive runs. The total distance (meters) covered represented the test result.

### Main Trial

On day 1, participants arrived to the laboratory at approximately 08:00 h after an overnight fast (> 10 h). Following a 15-min seated rest period and debrief, a cannula was inserted into an antecubital vein and a baseline venous sample was collected by a qualified phlebotomist. Participants were then given the high-fat meal, in the form of a Belgian chocolate ice cream (Haagen Daas) and whipping cream (Elmlea) shake with the meal being well tolerated by all participants. The meal quantity was adjusted relative to body mass, comprising of 1.2 g fat/kg, 1.2 g carbohydrate/kg, and 0.4 g protein/kg, being 17 ml of cream per 100 ml ice cream. A stopwatch was started when participants began consuming and the time taken to complete was recorded (mean time taken = 5.1 ± 2.2 min) and replicated in the subsequent trial. The stopwatch was reset and time taken for further blood samples again at 0.75, 2, 4, and 6 h post-meal consumption. Water was available ad libitum during the first trial; the volume ingested was recorded and replicated in the subsequent trial. Participants rested (reading, working quietly, watching television) throughout each observation period.

Participants were then randomly allocated to either 1 day (1FOOT; day 7) or 3 days (3FOOT; days 5, 6, 7) of football matches. All matches for both groups consisted of 60-min (2 × 30 min) small-sided game (5 vs 5) performed on an artificial outdoor court (36.5 × 27.5 m). In order to provide equal numbers for the matches, two additional participants were included in the 3FOOT group, and three additional participants were included for the 1FOOT. These additional five participants were not included in the analyses due to unavailability for testing. Two of these participants from each team played the additional (2 × 5 min of each half) turn in goal to meet the allotted time. All football matches were played at the same time of day (20:00 h) and each player in turn played (5 min) as goalkeeper for the matches. On day 8, they returned to the laboratory and repeated the high-fat meal and blood sampling procedure.

Participants were requested to complete a food diary for the 3 days before the initial trial and to replicate for the subsequent trial, as well as maintain their habitual dietary intake for the 4 days interim between these trials. To avoid the ingestion of excessive food intake, participants were allowed to consume a light snack (banana), at 21:30 h on the evening before the laboratory visits, but were instructed to keep this consistent for both trials. Before leaving the training facility, participants were reminded that they could drink plain water but should not consume any further food or drinks. Their dietary program the evening before the training was checked upon their arrival for the blood testing on day 8.

### Energy Expenditure and Match Load

The energy expenditure during the football matches was estimated from an Actigraph activity monitor (Actigraph, wGT3X-BT) placed on the hip [15] and estimated using the Freedson vector magnitude equation [16]. Session rate of perceived exertion (sRPE) was recorded within 5 min of match completion using the CR-10 Borg scale and player load calculated (sRPE × min) [17]. Perceived recovery status (PRS) was assessed on a scale of 0 (very poorly recovered/extremely tired) to 10 (very well recovered/highly energetic) approximately 10 min prior to each session [18, 19].

### Analytical Procedures

Blood samples were collected into 9-ml potassium-EDTA Monovettes (Sarstedt, Leicester, UK) or serum separate tubes (SST)-gel/clot activator. Serum was obtained by centrifugation (15 min at 1500*g* and at room temperature) and stored at − 80 °C for further analysis. Analyses of all samples from the same participant were done in a single batch. The spectrophotometric analyses were performed on an automated clinical chemistry system (Dimension Clinical Chemistry System-Xpand) to determine the level of TG, high-density lipoprotein (HDL), low-density lipoprotein (LDL), total cholesterol, insulin, and glucose and for complete blood count (Abbott-Cell Dyn 3700 Analyser), using appropriate reagents, calibrators, and controls. Highly sensitive serum C-reactive protein (hsCRP) (ng/ml), non-esterified fatty acids (NEFA; mmol l^−1^), and interleukin 6 (IL-6) concentrations (pg/ml) were determined from ELISA using a commercial kit (Elabscience, WA, USA), an automatic ELISA microplate reader (Infinite® 200 PRO NanoQuant, Switzerland) and Magellan Standard software (version 7.1). The limit of sensitivity was ≤ 0.01 ng/ml for CRP and 0.04 pg/ml for IL-6.

### Calculations

Triglyceride, glucose, and insulin responses were assessed as the total area under the curve (AUC) and incremental areas under the concentration-versus-time curves (iAUC) calculated using the trapezoidal rule. Incremental area under the curve was calculated by subtracting, from the postprandial area, the baseline value extrapolated over 6 h, thus reflecting changes occurring after the meal.

Whole-body insulin sensitivity was assessed with the homeostatic model assessment (HOMA) of insulin resistance index from serum fasting data as insulin (μU/ml) × glucose [(mmol/l)/22.5] [20] and with the Matsuda’s composite whole-body insulin sensitivity index (ISI) in the postprandial state as 10,000/square root of [fasting glucose (mg/dl) × fasting insulin (μU/ml) × mean postprandial glucose (mg/dl) × mean postprandial insulin (μU/ml)] [21]. Two-hour AUC and iAUC for insulin and glucose were chartered as determinants of insulin resistance [21].

### Statistical Analyses

Data were tested for normality using a Kolmogorov-Smirnov test, with all data shown to be normally distributed. A 2 × 2 × 5 [group (1FOOT, 3FOOT) × condition (pre- and post-match) × time points] analysis of variance (ANOVA) was used, and a Bonferonni post hoc test was applied when a significant interaction effect was found. Fasting concentrations, (incremental) area under the curve values, estimated energy expenditure, and subjective measures (sRPE and PRS) in the two different groups were compared between trials using Student’s *t* tests. *t* test for independent samples was used for the comparison between the groups and *t* test for dependent samples for the comparison of pre- to post-responses. Statistical significance was set at *p* < 0.05, and 95% confidence intervals (CI) and Cohen’s *d* effect sizes (ES) were calculated. The criteria to interpret the magnitude of the ES were as follows: > 0.2 small, > 0.6 moderate, > 1.2 large, and > 2.0 very large) [22]. All statistical analyses were performed with Statistical Package for the Social Sciences (SPSS), version 21.0. Descriptive statistics of the data are presented as means ± standard deviation (SD) unless otherwise stated.

## Results

### Energy Expenditure

There were no significant between-group differences for demographic and preliminary baseline measures (Table 1). There was no difference between 1FOOT and 3FOOT in the estimated energy expenditure during the last session (387 vs 347 kcals). The 1FOOT performed 122% greater amount of time in vigorous activity (20 vs 9 min) (*p* < 0.05; 95%CI = 0.3 to 21.0; ES = − 1.18) compared to the 3FOOT. For the 3FOOT condition, there were no differences in energy expenditure between sessions 1, 2, and 3 (350, 363, and 347 kcals, respectively). Also, no difference was found in sRPE between 1FOOT (325.7 ± 83.8 a.u.) and the third session of 3FOOT (400 ± 67.0 a.u.). For the 3FOOT, sRPE was 15.6% (*p* < 0.05; 95%CI = − 115.4 to − 4.6; ES = − 0.60) and 19% (*p* < 0.001; 95%CI = − 132.0 to − 21.3; ES = − 0.92) higher for the second and third match, compared to the first one. In the 3FOOT condition, PRS scores decreased by 20% and 22% from the first to the second and third match, respectively (*p* < 0.001; 95%CI = 1.10 to 2.44; ES = 3.60 and *p* < 0.01; 95%CI = 0.68 to 2.76; ES = 1.72, respectively).

### Triglycerides

**Fig. 2:**
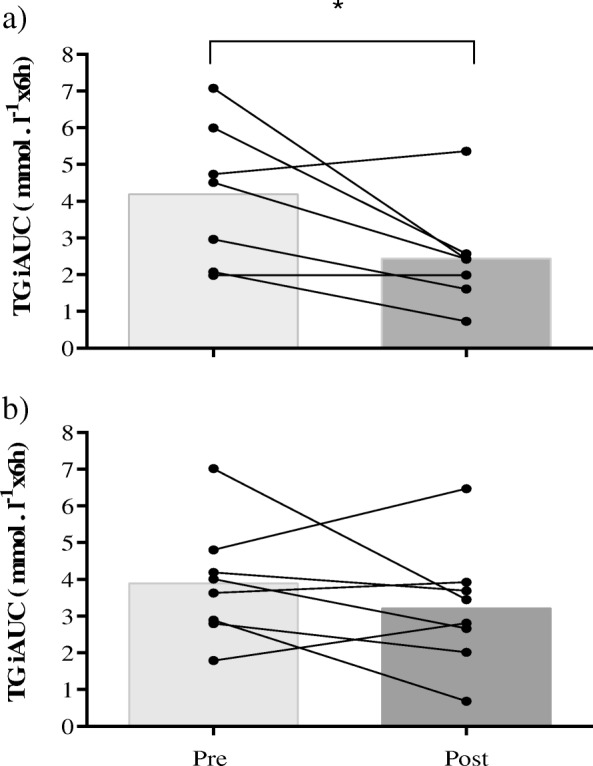
Individual and mean postprandial TG incremental area under the curve after high-fat meal test pre and post one **a** and three **b** 60-min football matches (*p < 0.05)

**Fig. 3:**
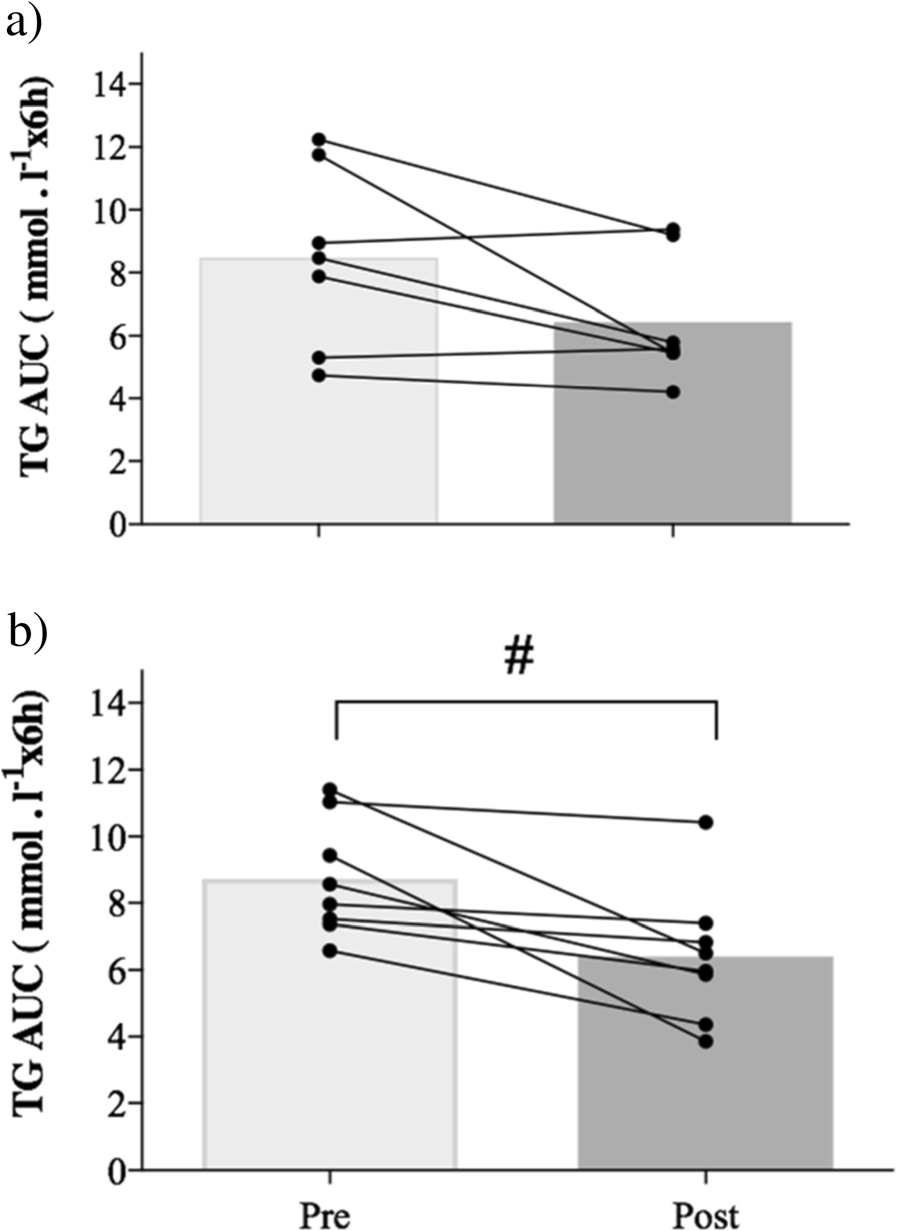
Individual and mean postprandial triglyceride total area under the curve (AUC) after high-fat meal test pre and post for **a** one and **b** three 60-min football matches (^#^*p* < 0.01)

**Fig. 4:**
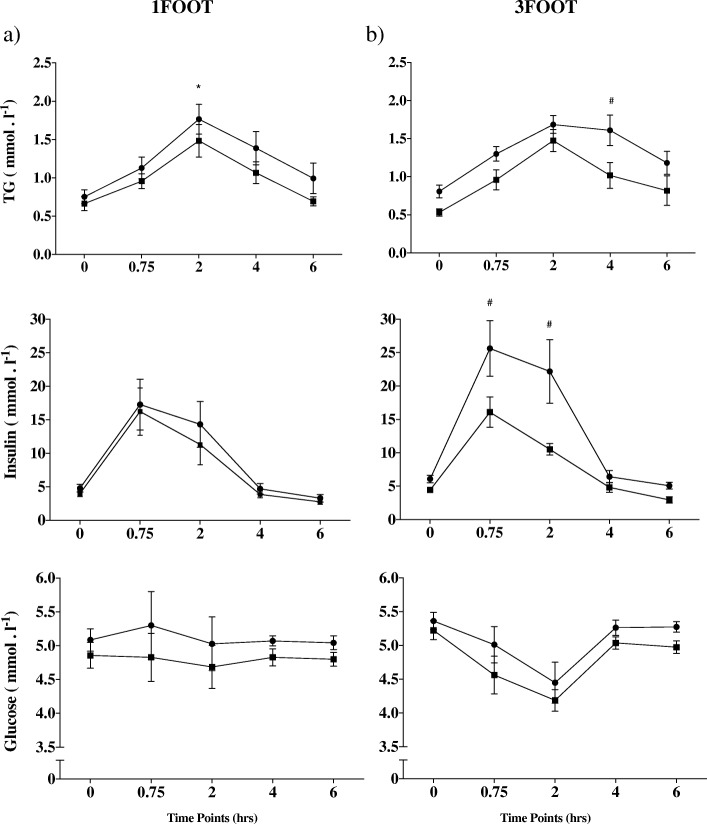
Fasting and postprandial time course from the high-fat meal after one (left column) and three consecutive (right column) 60-min football matches. Values are means SE. Circles are for pre-match and squares for post-match. *p < 0.05 between pre- and post-measures; ^#^*p* < 0.01 between pre- and post-measures

There was no difference between 1FOOT and 3FOOT for postprandial TG measures on day 1. Postprandial TG incremental area under the curve was 41% lower from pre- to post-trials for 1FOOT (*p* < 0.05; 95%CI = − 3.45 to − 0.04; ES = 1.02) and 15.7% lower for 3FOOT (ns; ES = 0.41) (Fig. 2). The postprandial TG total area under the curve was 24% (ns) lower in 1FOOT, whereas it was 26% lower from pre- to post-trial in 3FOOT (*p* < 0.01; 95%CI; − 3.96 to − 0.69; ES = 1.23) (Fig. 3). There was a decrease for postprandial TG at 2 h from pre- to post-exercise (*p* < 0.05; 95%CI 0.00 to 0.94; ES = 0.65) in 1FOOT. For the 3FOOT, there was a decline in postprandial TG at 4 h from pre- to post-matches (*p* < 0.001; 95%CI = 0.23 to 1.18; ES = 1.3) (Fig. 4).

### Glucose, Insulin, and NEFA

Postprandial insulin total and incremental AUC were lower at 0.75 h (*p* < 0.001; 95%CI = 3.25 to 16.94; ES = 0.99) and 2 h (*p* < 0.001; 95% CI = 5.78 to 19.47; ES = 1.39) (Table 2) for post- compared to pre-measures for the 3FOOT. There was a decrease between 2 h ISI for both the 3FOOT (*p* < 0.05; 95%CI = 0.96 to 9.28; ES = − 0.82) and 1FOOT (*p* < 0.05; 95%CI = 0.152 to 6.419; ES = − 0.51) conditions while only for the 3FOOT at the 6 h time point (*p* < 0.01; 95%CI = 1.853 to 11.05; ES = − 0.96) for post-matches. No time or condition differences were observed for NEFA, total cholesterol, HDL, or LDL (Table 3).

**Table 2:** Baseline blood lipids concentration (fasted), area under the curve (AUC), and incremental area under the curve (iAUC) for both the 1FOOT and 3FOOT at day 1 (pre) and day 8 (post). Data presented as mean (SD)

	Group	Pre	Post	% change from pre to post
TC (mmol l^-1^)	1FOOT	4.58 (0.29)	4.56 (0.67)	− 0.4
	3FOOT	5.22 (0.26)	5.02 (0.48)	− 3
HDL (mmol l^−1^)	1FOOT	1.40 (0.07)	1.50 (0.24)	7
	3FOOT	1.52 (0.12)	1.54 (0.10)	1
LDL (mmol l^−1^)	1FOOT	3.03 (0.26)	3.32 (0.81)	9
	3FOOT	3.54 (0.19)	2.88 (0.49)	18
HOMA	1FOOT	1.04 (0.43)	0.81 (0.28)	22
	3FOOT	1.45 (0.41)	1.02 (0.21)^#^	29
Matsuda ISI 2 h	1FOOT	14.86 (6.11)	18.14 (6.57)*	22
	3FOOT	11.20 (6.90)	16.33 (5.37)*	45
Matsuda ISI 6 h	1FOOT	18.06 (6.65)	21.83 (7.57)*	20
	3FOOT	13.16 (7.09)	19.61 (6.27)^#^	49
2 h insulin AUC (mmol l^−1^ 2 h^−1^)	1FOOT	29.06 (11.47)	24.79 (11.66)	− 14
	3FOOT	45.59 (25.81)	24.32 (6.80)*	− 47
2 h insulin iAUC (mmol l^−1^ 2 h^−1^)	1FOOT	22.45 (11.70)	18.13 (12.92)	− 19
	3FOOT	33.45 (23.64)	15.48 (5.93)*	− 54
2 h glucose AUC (mmol l^−1^ 2 h^−1^)	1FOOT	10.27 (2.04)	9.60 (1.55)	− 6
	3FOOT	9.82 (1.20)	9.13 (1.10)	− 7
2 h glucose iAUC (mmol l^−1^ 2 h^−1^)	1FOOT	0.18 (1.37)	− 0.12 (0.93)	− 166
	3FOOT	− 0.92 (1.29)	− 1.32 (0.87)	− 43

*TC* total cholesterol, *HDL* high-density lipoproteins, *LDL* low-density lipoprotein, *ISI* Insulinogenic Index, *HOMA* homeostatic model of assessment, *AUC* total area under the curve, *iAUC* incremental area under the curve, *1FOOT* one football session, *3FOOT* three consecutive football sessions

*p < 0.05 pre- vs post-measures; ^#^*p* < 0.01 pre- vs post-measures

**Table 3:** Blood concentrations for NEFA and acute-phase reactant proteins and inflammatory markers across all time points for both the 1FOOT and 3FOOT at day 1 (pre) and day 8 (post). Data are presented as mean (SD)

Measure	Group	Baseline		0.75 h		2 h		4 h		6 h	
		Pre	Post	Pre	Post	Pre	Post	Pre	Post	Pre	Post
NEFA (mg/dL)	1FOOT	*2.81 (0.93)*	*3.02 (1.53)*	*2.98 (1.19)*	*2.98 (1.11)*	*3.00 (1.10)*	*3.05 (1.15)*	*2.94 (1.24)*	*3.11 (1.09)*	*3.16 (1.37)*	*3.66 (1.72)*
	3FOOT	3.40 (1.03)	3.20 (0.78)	3.19 (0.86)	3.62 (0.57	3.08 (0.95)	3.27 (0.95)	3.31 (0.99)	3.31 (1.05)	3.76 (1.11)	3.51 (1.31)
hs-CRP (ng/ml)	1FOOT	2524.2 (2943.1)	2907.4 (3304.2)	1978.8 (2899.2)	3452.8 (3084.7)	2060.3 (3148.1)	3430.9 (3345.4)	2165.0 (3399.0)	3352.5 (2985.4)	1827.5 (2909.5)	3860.2 (3402.6)
	3FOOT	2529.0 (3715.7)	1499.3 (1122.2)	1105.3 (1948.6)	1402. 7 (1274.6)**	2499.6 (3490.2)	1491.1 (1158.5)**	2509.3 (3433.3)	1644.4 (1117.4)**	2454.3 (3512.9)	3869.2 (3246.7)
IL-6 (pg/ml)	1FOOT	0.783 (1.05)	1.97 (1.69)	0.85 (0.92)	1.83 (1.69)	0.843 (0.659)	1.63 (0.91)	1.39 (1.13)	1.9 (1.95)	1.89 (1.85)	2.46 (2.17)
	3FOOT	2.00 (2.08)	0.760 (0.45)	1.65 (2.32)	0.46 (0.39)	2.26 (2.93)	0.547 (0.274)*	3.47 (3.8)**	1.49 (1.52)*	3.50 (3.34)**	1.054 (0.769)*
Lymphocytes (× 10^9^/l)	1FOOT	2.07 (0.56)	2.01 (0.67)	1.82 (0.44)	1.88 (0.55)	1.98 (0.57)	1.88 (0.55)	2.39 (0.61)	2.27 (0.62)	2.42 (0.63)	2.65 (0.77)
	3FOOT	2.04 (0.36)	2.52 (0.61)*	1.84 (0.38)	1.97 (0.42)	1.92 (0.38)	2.07 (0.39)	2.23 (0.53)	2.57 (0.64)	2.10 (0.88)	2.84 (0.54)*
Neutrophils (× 10^9^/l)	1FOOT	3.00 (0.91)	3.81 (1.13)*	3.25 (0.89)	3.87 (1.05)*	3.50 (0.75)	3.90 (0.99)	3.62 (0.79)	3.91 (1.03)	3.84 (0.76)	3.97 (0.97)
	3FOOT	3.14 (1.05)	2.75 (0.40)**	3.62 (1.36)	2.77 (0.60)*,**	3.58 (1.36)	2.82 (0.61)*,**	3.82 (1.50)	2.81 (0.78)*,**	3.98 (1.62)	2.80 (0.86)*,**
Monocytes (× 10^9^/l)	1FOOT	10.28 (1.92)	10.21 (1.79)	9.41 (1.174)	8.95 (1.78)	9.37 (1.60)	10.35 (1.67)	9.21 (2.14)	9.94 (2.02)	8.55 (1.55)	9.38 (2.02)
	3FOOT	8.00 (0.80)**	8.95 (0.66)	7.58 (1.28)**	8.67 (1.05)	8.04 (1.34)**	8.81 (0.89)**	7.02 (2.18)**	8.30 (0.90)**	6.62 (2.38)**	7.47 (1.51)**

*hsCRP* = highly sensitive C-reactive protein, *IL-6* =  interleukin 6, *NEFA* = non-esterified fatty acids

*p < 0.05 from pre measures; **p < 0.05 from 1FOOT

### Inflammatory Markers

In the 3FOOT condition, post-IL6 were lower at 2 h (*p* < 0.05; 95%CI = − 3.30 to − 0.13; ES = 0.82), 4 h (*p* < 0.01; 95%CI = − 3.56 to − 0.39; ES = 0.69), and 6 h (*p* < 0.001; 95%CI = − 4.03 to − 0.86; ES = 1.01) compared to pre-measures, while no differences were observed for 1FOOT (Table 3). No within-group differences were shown for hsCRP at any of the time points; however, post-hsCRP were lower at 0.75 h (*p* < 0.005; 95%CI = 4.83 to 36.17; ES = 0.85), 2 h (*p* < 0.001; 95%CI = 373.3 to 3506; ES = 0.77), 4 h (*p* < 0.05; 95%CI = 141.6 to 3275; ES = 0.75), and 6 h (*p* < 0.001; 95%CI = 927.1 to 4060; ES = 1.00) in the 3FOOT compared to the 1FOOT condition.

Neutrophil count was lower at 0 h (*p* < 0.001; 95%CI = 0.33 to1.29 ES = 0.57) and 0.75 h (*p* < 0.01; 95%CI = 0.13 to 1.0 ES = − 0.68) for pre- compared to post-trial measures in the 1FOOT. For the 3FOOT, neutrophil count was lower for post- compared to pre-measures at 0.75 h (*p* < 0.001; 95%CI = − 1.33 to − 0.37; ES = 0.80), 2 h (*p* < 0.001; 95%CI = − 1.238 to − 0.2766; ES = 0.72), 4 h (*p* < 0.001; 95%CI = − 1.49 to − 0.53; ES = 0.84), and 6 h (*p* < 0.001; 95%CI = − 1.66 to − 0.70; ES = 0.90) (Table 3). No relationship between TG and IL6, hsCRP, or monocyte concentration were observed for any of the time points in both conditions.

## Discussion

The main finding of the present study was that a group performing a single 60-min recreational football match lowered postprandial TG incremental area under the curve with no further effect compared to a group performing three football matches on consecutive days. Our finding is in accordance with the observation no greater benefit of consecutive days’ of exercise on postprandial TG reduction, compared to one sole session [14, 23]. Such findings suggest there is no dose-response effect for accumulating consecutive days of exercise on lowering postprandial TG.

Although the exact mechanisms are unclear, the exercise-induced energy expenditure has often been purported as an important modulator for postprandial TG attenuation [12, 24]. Studies suggest that exercise-induced energy expenditure should be above a threshold of around 450 kcals to observe a beneficial postprandial effect, following a meal 10–12 h after the last exercise bout [25]. The notion being that a greater energy expenditure may reflect an increased TG clearance, which is facilitated by higher lipoprotein lipase (LpL) activity and, in turn, is stimulated by lowering of insulin. Conversely, elevated levels of insulin have shown to suppress muscle LpL and thus impair the hydrolysis of triglyceride-rich lipoproteins [26]. This may have been the case in our study given the carbohydrate composition of our meal and the elevated insulin levels at 0.75 and 2 h in the 3FOOT pre-match group. However, Peddie et al. [27] reported the effect of energy expenditure is predominantly on fasted, rather than postprandial TG measures, and may explain why some studies, including ours, have reported postprandial TG attenuation below the proposed threshold (~ 450 kcal). A relatively low exercise-induced energy expenditure for the last session of the 3FOOT group (387 vs 347 kcals; 1FOOT), coupled with the difference in high-intensity effort as well as the small evening snack (banana) consumed post-exercise may denote that the energy deficit was insufficient to cause a clear reduction in postprandial TG in the 3FOOT. Evidence shows that when the energy deficit is immediately replaced, the postprandial TG attenuation is no longer observed [13]. Therefore, it seems that an energy deficit, rather than energy expenditure per se, is likely to be more important in the attenuation of postprandial TG.

The reduction of postprandial TG incremental area under the curve in the 1FOOT in the present study may be due to the 11 min of more vigorous activity performed compared to the 3FOOT (20 vs 9 min). Evidence shows as short as 3 min of high-intensity exercise to be potent enough to have benefits on metabolic profile [28], while Freese et al [23] demonstrated four 30 s all-out sprints reduced postprandial triglyceride concentration. Moreover, performing high-intensity exercise has shown to produce lower fasted and postprandial TG as well as elevated postprandial fat oxidation, compared to moderate intensity [29, 30]. A possible mechanism may be the contraction-induced increases in muscle LpL that are specific to fast-twitch fibers [31]. Subsequently, the high-intensity efforts may facilitate greater TG uptake by the exercised muscle fibers [31].

While we did not find a greater benefit to consecutive days’ exercise on postprandial TG attenuation, it is important to highlight that repeated daily exercise sessions would likely be advantageous in terms of lowering daily postprandial TG. Since an exercise session was performed each of the three consecutive days for the 3FOOT group, it is likely that a postprandial TG lowering the effect of exercise occurred on each day, and not just after the third day, as is the case for the 1FOOT. Regular exercise training may have beneficial effects on postprandial lipid metabolism through short term increased LpL activity [32], as well as on longer-term improvements on cardiovascular fitness and reductions in body fat mass [12].

The present study also showed a significant reduction of postprandial insulin at 0.75 and 2 h from pre- to post-measures in the 3FOOT group. While we acknowledge that pre-match insulin levels in the 3FOOT group were higher at these time points, our findings suggest a better control of glucose load post-match in the 3FOOT condition. Research has shown short periods of consecutive days of exercise training can increase insulin sensitivity [33] and glycemic variability [34]. In the study by Kirwan et al. [33], 7 days of 30 min cycling and 30 min treadmill walking at ~ 70% of maximal aerobic capacity resulted in improvements in insulin action in the absence of weight loss. These improvements include increased insulin sensitivity and responsiveness as well as enhanced suppression of hepatic glucose production [33]. Performing consecutive days of exercise may translate into an increase of muscle GLUT-4 expression leading to improved glucose uptake and higher insulin sensitivity [35]. If given an adequate training stimulus, it has been shown such a rapid increase in GLUT-4 expression may occur within 2 days of exercise [35].

In a recent study, Steenberg et al. [36] found that exercise training history diminishes the ability of a single bout of exercise to enhance insulin sensitivity, albeit not specifically pertaining to the postprandial response. Nonetheless, while our participants were not “trained” and had ceased any significant physical activity for 1 week before the initial trial, they did have experience playing football. Steenberg et al. [36] reported that exercise training increases insulin-stimulated glucose uptake of skeletal muscle at rest, but with no further gains in the acutely exercised leg. Thus, the elevated levels of glycogen in muscle after training might reduce the ability of acute exercise to enhance insulin-stimulated glucose uptake through an attenuation of activated protein kinase activity and may be applicable in our findings.

Elevated postprandial TG and glucose have shown to play an important role in low-grade inflammation, via various cytokines and molecular pathways that precede the development of atherosclerosis [37–40]. We found interleukin 6 and neutrophil content generally to be lower for post- compared to pre-measures in the 3FOOT, although we found no association between changes in postprandial TG incremental area under the curve and any inflammatory markers. In the 3FOOT group, monocyte count was lower compared to the 1FOOT at most points pre- and post-exercise. Though consuming a high-fat meal has shown to induce a transient increase in reactive oxidative stress, due to activation of nuclear kappa factor [41], exercise has shown to reduce basal levels of systemic inflammation and oxidative stress [42]. Although direct comparisons with other studies are difficult, research has shown that three consecutive days of exercise may reflect a reduction in the production of reactive oxidative species, markers of oxidative stress, and an upregulation of antioxidant defense mechanisms in a cohort of highly trained cyclists [43]. However, there was no cumulative effect on the oxidation of lipids, and it is unlikely that this will directly influence postprandial TG; nonetheless, further research is warranted.

## Conclusion

In conclusion, the main finding of the present study was that a group performing a single 60-min recreational football match lowered postprandial TG incremental area under the curve with no further effect compared to a group performing three football matches on consecutive days. Such findings suggest there is no dose-response effect for accumulating consecutive days of exercise on lowering postprandial TG. This is important to better understand the short-term effects of regular exercise.

## Data Availability

Please contact the author for data requests.

## Abbreviations

1FOOT: One football match
3FOOT: Three football matches
AUC: Total area under the curve
95% CI: 95% confidence intervals
ES: Effect sizes
GLUT-4: Glucose transporter type 4
HDL: High-density lipoprotein
HOMA: Homeostatic model assessment
HR: Heart rate
hsCRP: Highly sensitive C-reactive protein
iAUC: Incremental area under the curve
IL-6: Interleukin 6
ISI: Insulin sensitivity index
LDL: Low-density lipoprotein
LpL: Lipoprotein lipase
NEFA: Non-esterified fatty acids
PRS: Perceived recovery status
sRPE: Session rate of perceived exertion
TG: Triglyceride
YYIETL1: Yo-Yo Intermittent Endurance Test Level 1
